# GW-2974 and SCH-442416 modulators of tyrosine kinase and adenosine receptors can also stabilize human telomeric G-quadruplex DNA

**DOI:** 10.1371/journal.pone.0277963

**Published:** 2022-12-07

**Authors:** Alaa A. Salem, Ismail A. El Haty, Mohammad A. Ghattas

**Affiliations:** 1 Department of Chemistry, College of science, United Arab Emirates University, Al Ain, United Arab Emirates; 2 College of Pharmacy, Al Ain University, Abu Dhabi, United Arab Emirates; D Y Patil Deemed To Be University, INDIA

## Abstract

GW-2974 is a potent tyrosine kinase receptor inhibitor while SCH-442416 is a potent adenosine receptors’ antagonist with high selectivity towards human adenosine A_2A_ receptor over other adenosine receptors. The two compounds were reported to possess anti-cancer properties. This study aimed to investigate whether stabilization of human telomeric G-quadruplex DNA by GW-2974- and SCH-442416 is a plausible fundamental mechanism underlying their anti-cancer effects. Human telomeric G-quadruplex DNA with sequence AG_3_(TTAGGG)_3_ was used. The study used ultraviolet-visible (UV-Vis), fluorescence, fluorescence quenching, circular dichroism (CD), melting temperatures (T_m_) and molecular docking techniques to evaluate interactions. The results showed that GW-2974 and SCH-442416 interacted with G-quadruplex DNA through intercalation binding into two types of dependent binding sites. Binding affinities of 1.3 × 10^8^–1.72 × 10^6^ M^−1^ and 1.55 × 10^7^–3.74 × 10^5^ M^−1^ were obtained for GW-2974 and SCH-442416, respectively. An average number of binding sites between 1 and 2 was obtained. Additionally, the melting temperature curves indicated that complexation of both compounds to G-quadruplex DNA provided more stability (ΔT_m_ = 9.9°C and 9.6°C, respectively) compared to non-complexed G-quadruplex DNA. Increasing the molar ratios over 1:1 (drug:G-quadruplex) showed less stabilization effect on DNA. Furthermore, GW-2974 and SCH-442516 have proven ≥ 4.0 folds better selective towards G-quadruplex over double-stranded ct-DNA. *In silico* molecular docking and dynamics revealed favorable exothermic binding for the two compounds into two sites of parallel and hybrid G-quadruplex DNA structures. The results supported the hypothesis that GW-2974 and SCH-442416 firmly stabilize human telomeric G-quadruplex DNA in additions to modulating tyrosine kinase and adenosine receptors. Consequently, stabilizing G-quadruplex DNA could be a mechanism underlying their anti-cancer activity.

## 1. Introduction

GW-2974, N4-(1-benzyl-1H-indazol-5-yl)-N6,N6-dimethyl-pyrido-[3,4-d]-pyri-midine-4,6-diamine, is a quinazoline derivative synthesized by David W. Rusnak [1] (Scheme 1). It is a tyrosine kinase inhibitor that is structurally analogous to the GW-572016 (also known as lapatinib), which was approved in 2007 by the Food and Drug Administration for treating breast cancer [2,3]. Additionally, GW-2974 at low concentrations is a dual inhibitor of EGFR and HER2 in glioblastoma multiforme *in vitro* and *in vivo* [3]. It explicitly inhibits EGFR and ERBB2 tyrosine kinase receptors in tumor cells, showing better therapeutic efficacy and limited toxicity in gallbladder and breast cancers than GW-572016 [1]. Furthermore, testing the effect of GW-2974 on gallbladder carcinoma in BK5.erbB2 mice revealed that targeting Egfr alone or in combination with Erbb2 was effective in preventing and treating gallbladder carcinoma in BK5.erbB2 transgenic mice [4]. GW-2974 was also found to prevent oral carcinogenesis induced by 7,12-dimethylbenz[**a**]anthracene (DMBA) in hamster cheek pouch model at the post-initiation stage in part by suppressing aberrant AA metabolism [5].

Scheme 1. Chemical structures of GW-2974, SCH-442416, G-quartet and G-quadruplex DNA.



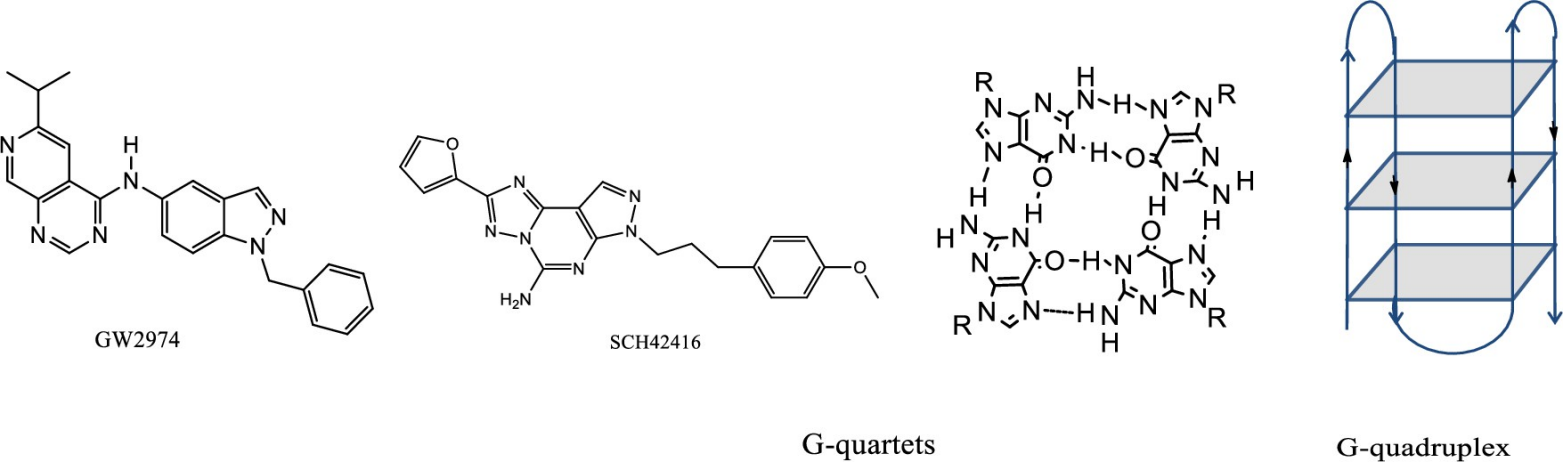



The combination of INCB3619, a potent inhibitor of ADAM10 and ADAM17, with GW-2974, a dual inhibitor of EGFR and HER-2/neu kinases, resulted in a synergistic growth inhibitory effect on MCF-7 and HER-2/neu-transfected MCF-7 human breast cancer cells [6]. Additionally, the use of the combination of GW-2974 with a MET inhibitor, yielded maximum growth inhibition in the treatment of non-small cell lung carcinoma (NSCLC) than the use of any individual inhibitor [7]. Furthermore, the combination of GW-2974 with GX15-070 or HA14-1 inhibitors of Bcl-2 was found to exert a synergistic growth inhibitory effect on MCF-7, MCF/18 and MTR-3 human breast cancer cell lines [8]. Oral administration of GW-2974 was also found to effectively inhibit skin tumor’s progression in BK5.erbB2 and wild-type mice [9].

Chen et al. studied the interaction of GW-2974 with ABC transporters and reported that GW-2974 reverses ABCB1- and ABCG2-mediated multidrug resistance by blocking their efflux function. It was concluded that the combination of GW-29714 with EGFR tyrosine kinase inhibitors is beneficial for treating cancer [10].

GW-2974 was also found to carry out cardiac cell protective activity by preventing apoptosis induced by TNFα, a known cytokine detected in cardiac failure [11]. Moreover, GW-2974 was reported to have inhibitory effects on hepatitis C virus activity and PC-3 cell growth. It also stimulated neuron-specific enolase and chromogranin in an androgen-independent prostate cancer cell line PC-3 [12,13].

SCH-442416, 5-amino-7-[3-(4-methoxy)phenylpropyl]-2-(2-furyl)-pyrazolo [4,3-e]-1,2,4-triazolo[1,5-c]pyrimidine (Scheme 1), is a pyrazolo-triazolo-pyrimidine derivative synthesized by Baraldi as a highly potent and selective human adenosine A_2A_ receptor antagonist [14,15]. The role of SCH-442416 during the proliferation and osteogenic differentiation of human primary bone marrow stromal cells was examined. The treatment of HPOC cultures with SCH-442416 induced osteogenic differentiation in CPA, resulting in increased alkaline phosphatase activity [16]. Several derivatives of SCH-442416 have been prepared with an aim to enhance its potency and selectivity [17–21].

Furthermore, the effect of L-DOPA combined with SCH-442416 on the rotational behaviors observed in a hemi-Parkinson mouse induced by unilateral 6-hydroxydopamine (6-OHDA) injection was investigated. SCH-442416 was found to reduce L-DOPA dosage in the 6-OHDA-induced hemi-Parkinson mouse model [22]. Zhao et al. also reported that lower concentrations of SCH-442416 may increase the mRNA expression of the Kir 2.1 and Kir 4.1 channels in Müller cells. Subsequently, it may accelerate K^+^ removal to protect the retinal neurons *in vitro* under hypoxic conditions [23,24]. Zhong et al. also investigated the effect of SCH-442416 on the expression of glutamine synthetase (GS) and glutamate aspartate transporter (GLAST) in rat retinal Müller cells at increased hydrostatic pressure *in vitro*. SCH-442416 was found to increase the expression of GLAST and GS in Müller cells, consequently accelerating the removal of extracellular glutamate [25].

On the other hand, G-quadruplex DNA structures are formed by the guanine-rich strands in human telomeric chromosomes; the immunoglobulin switch and the gene promoter-binding regions of *c-myc*, *k-ras* and *c-kit*, under physiological conditions.

Single or multiple strands of DNA can fold into G-quadruplex structures through eight intra- or inter-molecular Hoogsteen hydrogen bonds. Every four guanines in a plane form a layer called G-tetrads or G-quartets where the Watson–Crick edge of a guanine base forms two hydrogen bonds with the Hoogsteen edge of a neighboring guanine base. G-quartets are held together by *π*–*π* stacking to form the quadruplex structure and stabilized by electrostatic interactions between the guanine carbonyl groups and alkali metal cations as shown in Scheme 1 [26–30].

Telomeres are the endcaps of chromosomes. They do not contain genetic information and consist of 5–15 kb of double-stranded DNA and 100–300 nucleotide bases at the single-stranded 3′ end [31]. Interestingly, cell division capability is linked to telomere length. In normal somatic cells, the telomere is shortened by 50–200 nucleotides during each cell division and reaches a critical length at which it eventually enters the senescence stage. Subsequently, the cells undergo apoptosis [32]. Telomerase enzyme re-elongates the telomeres in stem, germ and embryonic cells. The telomerase enzyme in cancer cells maintains telomere length, enabling the cells to undergo indefinite divisions [33]. Folding the telomere’s single-stranded overhang into G-quadruplex DNA conceals the telomere end that acts as a template/substrate for RNA telomerase, thereby inhibiting telomerase activity. It also displaces telomere-binding proteins involved in telomere capping, which results in the cells to identify the folded telomere as a region of DNA damage [34].

The stabilization of G-quadruplex structures via small molecules interferes with the biological function of telomerase by inhibiting its activity and altering its maintenance, which is crucial for cancer cells to divide indefinitely [34]. Subsequently, molecules with a preferential affinity towards G-quadruplexes can inhibit cancer proliferation. Therefore, this has become an attractive strategy for developing novel, effective and selective DNA-based anti-cancer agents [35–38].

Several small molecules were previously reported as G-quadruplex stabilizers, telomerase inhibitors and/or oncogene regulators [39]. Anthraquinones [40,41], acridines [42,43], porphyrins [44,45], porphyrazines [46], phtalocyanines [47] and telomestatin [48,49] were reported to bind to G-quadruplex DNA. Moreover, complexes of divalent selenium, manganese, nickel and copper ions with porphyrin, zinc with phthalocyanine and nickel with salphen showed high affinity and selectivity towards human telomeric and *c-myc* G-quadruplex DNA [30,31,50–52].

An aromatic planner core and a protonated side chain are important features of G-quadruplex DNA binders. The aromatic planner core intercalates between the G-quartets through π–π stacking while., the side chain support stability of formed complexes via hydrophobic or ionic interactions with the external side chains or into the DNA grooves [39,53,54]. For example, the stabilization of G-quadruplex by perylene was attributed to the length of the side chain linking the aromatic planner core to the protonated nitrogen atom and the basicity of the system [51,55]. For tetra(N-methyl-4-pyridyl) porphyrin, the porphyrin ring is assumed to π–π stack on the G-quartet, whereas the four lateral pyridinium groups are bound onto the grooves of G-quadruplex DNA [56]. Thus, an excellent G-quadruplex stabilizer must have an aromatic planar core to intercalate by π–π stacking between the G-quartets DNA planes and protonated side chains for face stacking and groove binding. A partial positive charge may also be required to substitute K^+^ or Na^+^ cations in the guanine’s center and bind to the negatively charged phosphate backbones [57].

This work aimed to study the stabilization of human telomeric G-quadruplex DNA (AG_3_(TTAGGG)_3_) caused by GW-2974 and SCH-442416 as a probable underlying mechanism for their anti-cancer effects. Interaction parameters such as binding affinity, binding constant, melting temperature (T_m_) and binding selectivity towards G-quadruplex over DNA duplex were studied using ultraviolet–visible (UV–Vis) spectroscopy, fluorescence and fluorescence quenching spectroscopy, circular dichroism (CD) spectroscopy, melting temperature and molecular docking techniques. The hypothesis that both GW-2974 and SCH-442416 stabilize human telomeric G-quadruplex DNA and consequently inhibit telomerase activity and cancer proliferation will be evaluated. This is in addition to their confirmed activities as tyrosine kinase and adenosine receptor modulators.

## 2. Materials and methods

### 2.1. Reagents

All chemicals were of the highest purity available (98%-99%) and used without further purification. GW-2974 (N4-(1-benzyl-1H-indazol-5-yl)-N6,N6-dimethyl-pyrido-[3,4-d]-pyrimidine-4,6-diamine), SCH-442416 (5-amino-7-[3-(4-methoxy) phenylpropyl]-2-(2-furyl)-pyrazolo[4,3-e]-1,2,4-triazolo[1,5-c]pyrimidine), ethylenediaminetetraacetic acid (EDTA), potassium chloride, Tris(hydroxymethyl)-aminomethane hydrochloride (Tris–HCl) and calf thymus DNA (c-DNA) were purchased from Sigma-Aldrich. High-performance liquid chromatography (HPLC)-purified telomeric DNA sequences, 5′-AGGGTTAGGGTTAGGGTTAGGG-3′, Fl-5′-AGGGTTAGGGTTAGGGTTAG-GG-3′ and 3′-TCCCAATCCCAATCCCAATCCC -5′, were purchased from Alpha DNA, Canada. Notably, Millipore deionized water was used in all experiments.

### 2.2. Apparatus

Absorption measurements were carried out using an Agilent-8453 UV–Vis spectrophotometer (Austria) and 1.0-cm quartz cells. Fluorescence measurements were performed in a 1.0-cm quartz cell using Cary Eclipse model-3 spectrofluorometer equipped with a high-intensity xenon flash lamp (Austria). Additionally, CD measurements were performed using the Jasco J-815 spectrophotometer (USA), and pH was measured using an Orion-401 Plus pH meter and Orion glass electrode.

### 2.3. Standard solutions

#### Buffer solutions

A 10^−2^ M Tris–KCl buffer solution (pH 7.4) was prepared by dissolving 10.0 mM Tris–HCl (1.576 g), 1.0 mM Na_2_EDTA (0.3722 g) and 100.0 mM KCl (7.455 g) in 1.0 L of deionized water. Then, the pH was adjusted using the glass electrode.

#### GW-2974 and SCH-442416 solutions

Stock solutions (2 × 10^−3^ M) of GW-2974 and SCH-442416 were prepared in ethylene glycol. Additionally, solutions of lower concentrations were prepared using appropriate dilutions of the buffer solution.

#### Calf thymus DNA (ct-DNA)

First, 1000.0 μg/mL ct-DNA was prepared by dissolving 10.0 mg of ct-DNA into 10.0 mL Tris–KCl buffer (pH 7.4) without sonication or stirring. Next, the solution was gently inverted overnight at 4°C to fully solubilize the DNA and prevent shearing of the large genomic DNA. The resulting solution was stable for several months at 4°C.

#### Human Telomeric Single-Stranded DNA

The purchased synthetic human telomeric DNA sequence 5′-AGGGTTAGGGTT-AGGGTTAGG G-3′, its fluorescein-labelled 5′ oligonucleotide 5′-Fl-AGGGTTAGGGTTAG-GGTTAGGG-3′ or its complementary strand 3′-TCCCAATCCCAATCCCAATC-CC-5′ were centrifuged for 10 min at 7000 rpm to obtain the DNA at the bottom of the tube. A 2.00 mL Tris–KCl buffer of pH 7.4 was added to each tube. The solutions were left overnight at 4°C for rehydration and then vortexed for 30 s. The reconstituted primers were stable for more than 6 months at 4°C.

To determine the concentration of resulting telomeric DNA solutions, a 10.0 μL were diluted into 1.0 mL using the Tris–KCl buffer, vortexed for 15 s, and its absorbances at 260 and 280 nm were measured. Concentration (in μg/mL) was calculated using the following equation:

$$\text{C}\left(\mu \text{g}/\text{mL}\right)=A_{260}\times \text{weight}\;\text{per}\;\text{OD}\times \text{dilution}\;\text{factor},$$

where OD is the optical density at 260 nm. The purity of each oligonucleotide was estimated based on the ratio *A*_260_/*A*_280_. Ratios ≥ 1.8 were considered enough to indicate high purity [58].

### 2.4. Procedures

#### Formation of human telomeric G-quadruplex DNA

The G-quadruplex structure was prepared by gently heating 2.0 mL of stock single-stranded 5′-AGGGTTAGGGTTAGGGTTAGGG-3′ DNA to about 95°C. Then, the solution was incubated for 10 min, left to cool down to room temperature and kept at 4°C until further use. Similarly, the fluorescein-labelled G-quadruplex DNA was prepared.

#### Hybridization of human telomeric single-stranded DNA oligonucleotides

First, a 1 × 10^−4^ M telomeric double-stranded DNA was obtained by mixing equimolar amounts of 5′-AGGGTTAGGGTTAGGGTTAGGG-3′ (268.8 μL of 7.44 × 10^−4^ M) with its complementary strand 3′-TCCCAATCCCAATCCCAATCCC-5′ (738.0 μL of 2.71 × 10^−4^ M). The volume of the solution was brought up to 2.0 mL using Tris–KCl buffer (pH 7.4). Next, the solution was vortexed for 15 s and incubated at 95°C for 10 min. The resulting hybridized double-stranded DNA was left at room temperature to cool down and kept in the refrigerator at 4°C until further use.

#### Spectrophotometric titrations

Absorption titrations were conducted by sequentially adding 10.0 μl aliquots of telomeric G-quadruplex (1.44 × 10^−4^ M) to 2.0 ml of GW-2974 or SCH-442416 (5 × 10^−5^ M) in a Tris–KCl buffer (pH 7.4). After each addition, the solution was shaken, incubated for 3 min at room temperature and scanned in the UV–Vis range of 200–700 nm. Additionally, increasing the incubation duration to 1 h was ineffective. Finally, solutions having ≤10% ethylene glycol were used during titration.

The titration was reversed by adding 10.0 μl aliquots of GW-2974 or SCH-442416 (1. × 10^−5^ M) to telomeric G-quadruplex (1.44 x 10^−4^ M).

For the fluorescence titration, 10 μl increasing concentrations of human telomeric G-quadruplex DNA (1.44 × 10^−4^ M) were added to 3.0 ml of GW-2974 or SCH-442416 (5 × 10^−6^ M) in Tris–KCl-buffer of pH 7.4. Then, the solution was stirred for 20 s, incubated for 3 min and scanned for its fluorescence spectra in the range of 300–550 nm. The titration was concluded when no alteration in the fluorescence intensity was observed. Notably, solutions having ≤ 10% ethylene glycol were maintained during the titration. Additionally, increasing the incubation period to 1 h was found to be ineffective. Excitation wavelengths of 243 and 268 nm were used for GW-2974 and SCH-442416, respectively, with an excitation and emission slit width of 10.0 nm. The stoichiometric ratios for drug/G-quadruplex was calculated using the molar ratio method through plots of fluorescence intensity versus drug/G-quadruplex molar ratio.

Moreover, in the fluorescence quenching titration, 10 μl aliquots of GW-2974 or SCH-442416 (1.0 x 10^−4^ M) were added in increasing concentrations to 3.0 ml of the fluorescein-labelled G-quadruplex (2 × 10^−6^ M) (5′-Fl-(AGGGTT)3AGGG-3′). Then, the solution was stirred for 20 s, incubated for 3 min and scanned for fluorescence at an excitation wavelength of 494 nm and emission wavelength of 518 nm. Finally, fluorescence quenching of fluorescein-labelled G-quadruplex was recorded using slit widths of 5.0 and 2.5 nm for excitation and emission, respectively.

The conformational changes in G-quadruplex DNA observed upon interactions of G-quadruplex with GW-2974 and SCH-442416 were also assessed using CD titrations. First, 10 μl increasing concentrations of either GW-2974 or SCH-442416 (5 × 10^−4^ M) were added to 1.0 ml of G-quadruplex DNA (4 × 10^−6^ M) in Tris–KCl buffer of pH 7.4. Then, the solution was shaken, incubated for 3 min at room temperature and CD spectra was scanned in the range of 200–400 nm at 50 nm/min and bandwidth of 1 nm using three accumulations. Furthermore, the obtained CD spectra were corrected for the blank and baseline. Furthermore, CD intensity changes at 293 nm were recorded in relation to ligand concentrations.

All UV-Vis, fluorescence and CD signals were corrected for the effect of dilution.

#### Melting temperature curves

Melting temperature (**T**_**m**_**)** curves for G-quadruplex DNA and its GW-2974 and SCH-442416 complexes were constructed using CD spectral measurements. First, a 1.0 ml solution of telomeric G-quadruplex DNA in Tris–KCl-buffer (3.93 × 10^−6^ M) was heated up to 95 °C in an average increment of 3 °C and average incubation time intervals of 3 min. Additionally, the CD spectra were recorded at each temperature in the 200–400 nm range using the same CD settings as mentioned in Section 2.4.3. Next, for constructing T_m_ curves for G-quadruplex DNA complexes with GW-2974 or SCH-442416, equimolar amounts of G-quadruplex DNA prepared in 1.0 mL KCl-Tris buffer (27.3 μL of 1.44 × 10^−4^ M) were mixed with GW-2974 or SCH-442416 (39.3 μL of 1 × 10^−4^ M). CD spectra of the resulting complexes were collected and corrected for blank and baseline. Lastly, plots of CD intensity at 293 nm versus temperature were constructed.

Similarly, the T_m_ curve of ct-DNA was obtained by heating 1.0 ml of ct-DNA in Tris–KCl-buffer of pH 7.4 (1 × 10^−9^ M, 100 ppm) at 25 °C–95 °C in average increments of 3.0°C applying an average incubation time intervals of 3 min. The intensity of the ct-DNA’s CD peak at 283 nm was recorded and plotted against temperature. The T_m_ curves of ct-DNA complexes with GW-2974 and SCH-442416 were made by mixing ct-DNA (100 μl, 1000 ppm, 8 × 10^−8^ M) with GW2974 or SCH442416 (23.4 μL, 1 × 10^−4^ M). The solutions’ volumes were brought up to 1.0 mL using Tris–KCl-buffer of pH 7.4, and CD intensities at 283 nm were recorded. About 10.0% of ethylene glycol was used for all measurements.

#### Selectivity towards telomeric G-quadruplex DNA

Binding selectivity of GW-2974 and SCH-442416 towards G-quadruplex DNA was assessed using telomeric double-stranded DNA and ct-DNA as interfering species.

First, a 5 × 10^−10^ M solution in each of fluorescein-labelled G-quadruplex DNA and GW-2974 or SCH-442416 was obtained by mixing suitable amounts of each into 2.0 mL Tris–KCl-buffer of pH 7.4. Then, the solution was vortexed for 10 s, incubated for 3 min and scanned for its fluorescence in the range of 500–700 nm using 494 nm as the excitation wavelength.

Additionally, appropriate amounts of telomeric double-stranded DNA or ct-DNA were added to the previous solutions to obtain solutions of 10.0, 50.0 and 100.0 concentration folds relative to fluorescein-labelled G-quadruplex DNA. The resulting solutions were vortexed for 10 s, incubated for 30 min at room temperature and scanned for their fluorescence in the range of 500–700 nm. In all solutions, 10% ethylene glycol was used.

#### Binding affinity

Binding affinities of GW-2974 and SCH-442416 towards telomeric G-quadruplex DNA were estimated using the Scatchard model. Fluorescence emissions obtained after adding different amounts of G-quadruplex DNA to constant amounts of GW-2974 or SCH-442416 were collected as described in section 2.4.3. Excitation wavelengths of 243 and 268 nm were respectively used for GW-2974 and SCH-442416.

In Scatchard equation $\frac{r}{C_{f}}$ is plotted versus *r* according to Eq 1 [59].

$$\frac{r}{C_{f}}=nK-Kr$$ (1)

where r is the mole ratio between bound ligand (*C*_*b*_) to DNA quadruplex, (*C*_*f*_) is the free concentration of ligand, *n* is the number of equivalent binding sites on G-quadruplex molecule and K is the binding constant. Then *C*_*b*_ was calculated using Eq 2

$$C_{b}=C_{total}-C_{f}$$ (2)

where *C*_*total*_ is the concentration of GW-2974 or SCH-442416 with no addition of quadruplex. *C*_*f*_ was calculated using Eq 3

$$C_{f}=C_{total}\left(1-\alpha \right)$$ (3)

where *α* is the fractionality factor and calculated using Eq 4.

$$\alpha =\frac{F_{f}-F}{F_{f}-F_{b}}$$ (4)

where *F*_*f*_ and *F*_*b*_ are the fluorescence of the free and fully bound ligand and *F* is the fluorescence at any given point during titration. Plotting $\frac{r}{C_{f}}$ versus *r* gives a slope equal to K and intercept equals *nK*. The plot can result in a straight or curved line. In the case of a curved line, therefore, Eq 1 is rearranged to Eq 5

$$r=\frac{nK\;C_{f}}{1+K\;C_{f}}$$ (5)

where r is plotted against *C*_*f*_. Values for n and K were obtained using non-linear curve fitting in Origin 8.0 software.

#### In silico molecular docking and dynamic simulation

*Molecular docking protocol*. Molecular dockings of GW-2974 and SCH-442416 onto telomeric G-quadruplex DNA were performed using the Glide software and the telomeric G-quadruplex DNA crystal structures PDB:2MS6 and PDB:6CCW from the protein data bank [60]. First, the structures were checked for missing atoms via the MOE software package [61]. Then, all solvent molecules and hetero-ligands were removed, and the structures were prepared for docking using the preparation wizard tool in the Maestro software [62–64]. Preparation included creating bonds, adding hydrogens, assigning partial charge for all atoms and checking the protonation state of each ionizable group. Next, binding pockets were identified from the quercetin and epiberberine’s co-crystallized ligands. Moreover, a grid box was produced for each, using the Receptor Grid Generation module in Glide [65]. On the other hand, GW-2974 and SCH-442416 molecules were prepared in Maestro [64] via the LigPrep module [66]. Ligand’s preparation included identifying the ionization states for ionizable functional groups at the pH range of 7.0 ± 2.0.

Subsequently, the two ligands were docked into the two prepared binding pockets on the G-quadruplex DNA structures using the Glide software [65]. Furthermore, the extra-precision (XP) docking mode [67] was used for conformational sampling. All produced poses were scored and ranked via the Glide-XP scoring function. The latter included terms for van der Waals, hydrogen bond, electrostatic interactions, desolvation penalty and penalty for intra-ligand contact [67].

*Molecular dynamics protocol*. The Telomeric G-quadruplex DNA complexes obtained by docking GW-2974 and SCH-442416 on PDB:6CCW were solvated and built separately via XLeap in the Amber Tools program. Both systems were energy minimized and simulated via pmemd in the Amber18 software package. Minimization was performed in two steps of 1000 cycles each; with applying 2 kcal/mol restraint forces on the proton atoms only in the first stage. The resultant systems were heated over the time course of 20 ps from 0 to 300 K under NVT conditions using Langevin thermostat and 2 kcal/mol restraint forces (on proton atoms). Subsequently, the solvated systems’ densities were equilibrated for 100 ps and using again 2 kcal/mol restrain forces on proton atoms. 100-ns MD simulations were conducted under NPT conditions with an average pressure of one atm and a relaxation time of 2 ps. The average temperature was fixed to 300 K, which was controlled by Langevin thermostat (with a collision frequency of 1.0 ps-1). The SHAKE algorithm was employed to constrain hydrogen atoms’ covalent bonds during the course of simulation. The Particle mesh Ewald algorithm [68] was employed to run all explicit solvent calculations, with a cutoff of 10 Å for long-range electrostatics.

## 3. Results and discussion

Interactions of GW-2974 and SCH-442416 with human telomeric G-quadruplex DNA were evaluated using UV–Vis spectroscopy, fluorescence emission, fluorescence quenching and CD. Additionally, binding parameters including binding constants, stoichiometries, binding modes, T_m_ and selectivity towards G-quadruplex over double-stranded DNA and ct-DNA were investigated. The data for the abovementioned investigations were supported by molecular docking.

### 3.1. Solubility of GW-2974 and SCH-442416

GW-2974 and SCH-442416 are insoluble in water but soluble in dimethyl sulfoxide and ethylene glycol. Moreover, GW-2974 and SCH-442416 solutions prepared using ethylene glycol (100%) were stable for more than four months at 4 °C. Stabilities of GW-2974 and SCH-442416 in 10% ethylene glycol and Tris–KCl buffer were tested using UV–Vis spectrophotometry. The solutions showed no significant changes in absorption values over 24 h. This time was considered enough to run all the experiments, and we used 10% ethylene glycol in all our measurements.

The effect of ethylene glycol on the structural conformation of telomeric G-quadruplex DNA was also tested. The addition of ethylene glycol to G-quadruplex (up to 20%.) in Tris–KCl buffer did not alter the shape and intensity of G-quadruplex’s CD spectrum. Therefore, these results indicate the safety of 20% ethylene glycol, which did not cause conformational changes in or denature telomeric G-quadruplex DNA. These results are consistent with those of Bonner and Klibanov’s report showing that 90% ethylene glycol was safe and did not denature DNA [69]. Therefore, freshly prepared 10% ethylene glycol buffer solutions were used throughout this study to improve the solubility of GW-2974 and SCH-442416.

### 3.2. Spectrophotometric titrations

Interactions of human telomeric G-quadruplex DNA with both GW-2974 and SCH-442416 were evaluated spectrophotometrically using UV-Vis absorption, fluorescence, and circular dichroism titrations as follows:

#### UV–Vis absorption

**Fig 1:**
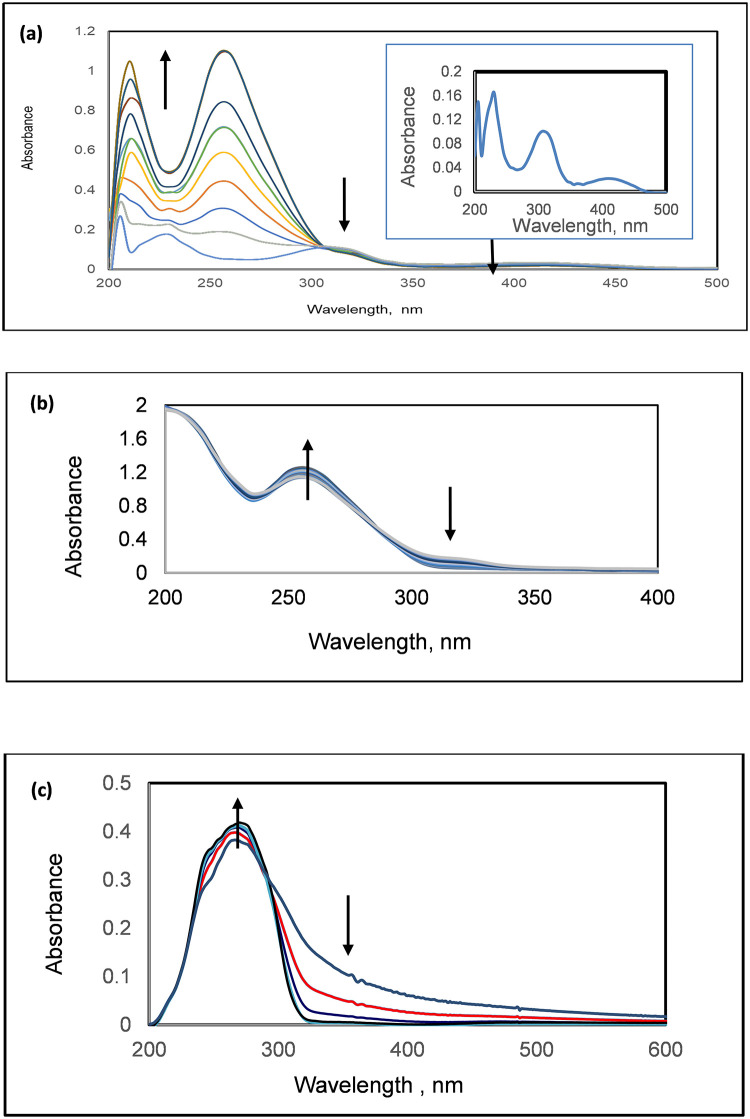
Spectrophotometric titrations of (a) A 5 × 10^−5^ M GW-2974 with 10.0 μl increments of human telomeric G-quadruplex DNA (1.44 × 10^−4^ M). (b) A 4.00 × 10^−6^ M human telomeric G-quadruplex DNA with 10.0 μl increments of GW-2974 (1 × 10^−5^ M). (c) A 5 × 10^−5^ M SCH-442416 with 5.0 μl increments of human telomeric G-quadruplex DNA (1.44 × 10^−4^ M). All titrations were carried out in Tris–KCl buffer, pH 7.4.

Fig 1 shows the UV–Vis absorption spectra of GW-2974 and SCH-442416 in Tris–KCl buffer (pH 7.4). In Fig 1a insert, the GW-2974 spectrum revealed strong absorption bands at 240 nm (ε = 6100) and 320 nm (ε = 5500) and a weak band at 420 nm (ε = 2600). On the other hand, the spectrum of SCH-442416 revealed a strong absorption band at 267 nm (ε = 7700) (Fig 1c).

Additionally, changing the pH of GW-2974 and SCH-442416 solutions (10^−5^ M) revealed no substantial spectral changes in the pH range 4.0–10.0. Therefore, these compounds were chemically stable within this pH range and could be safely used to further investigate their interactions with G-quadruplex DNA at the physiological pH of 7.4.

Fig 1a shows the effect of adding 10.0 μl increasing concentrations of human telomeric G-quadruplex DNA (1.44 × 10^−4^ M) to GW-2974 (5 × 10^−5^ M) solution prepared in Tris–KCl buffer (pH 7.4). At 420 and 320 nm, the absorption band intensity steadily decreased (hypochromicity) without forming isosbestic points or shifting. On the contrary, the absorption bands intensity at 240 nm increased in association with the increases in the DNA absorption band at 260 nm.

Fig 1b shows the effect of adding 10.0 μl increasing concentrations of GW-2974 (1.0 × 10^−5^ M) to G-quadruplex DNA (1.44 × 10^−4^ M) solution prepared in Tris–KCl buffer (pH 7.4). The absorption intensity of G-quadruplex DNA at 260 nm steadily increased due to the overlap with the GW-2974 at 240 nm. This is associated with slight decreases for the absorption intensity at 320 nm.

Fig 1c indicates that adding 25.0 μl increments of human telomeric G-quadruplex DNA (1.44 x 10^−4^ M) to SCH-442416 (5 x 10^−5^ M) increased the absorption band intensity at 267 nm and decreased it at 350 nm. Thus, the former can be attributed to interference between the absorption bands of G-quadruplex DNA and SCH at 260 nm and 267 nm, respectively.

The above-mentioned hypochromic effects (≥50%) observed on adding DNA to GW-2974 or SCH-442416 suggested the intercalation of both the compounds with telomeric G-quadruplex DNA. Hypochromicity along with binding constants and stoichiometry were correlated with intercalation binding modes via end-stacking on G-quartet and outside binding [56]. For obtaining further evidence, fluorescence and CD spectroscopic measurements were performed.

#### Fluorescence

**Fig 2:**
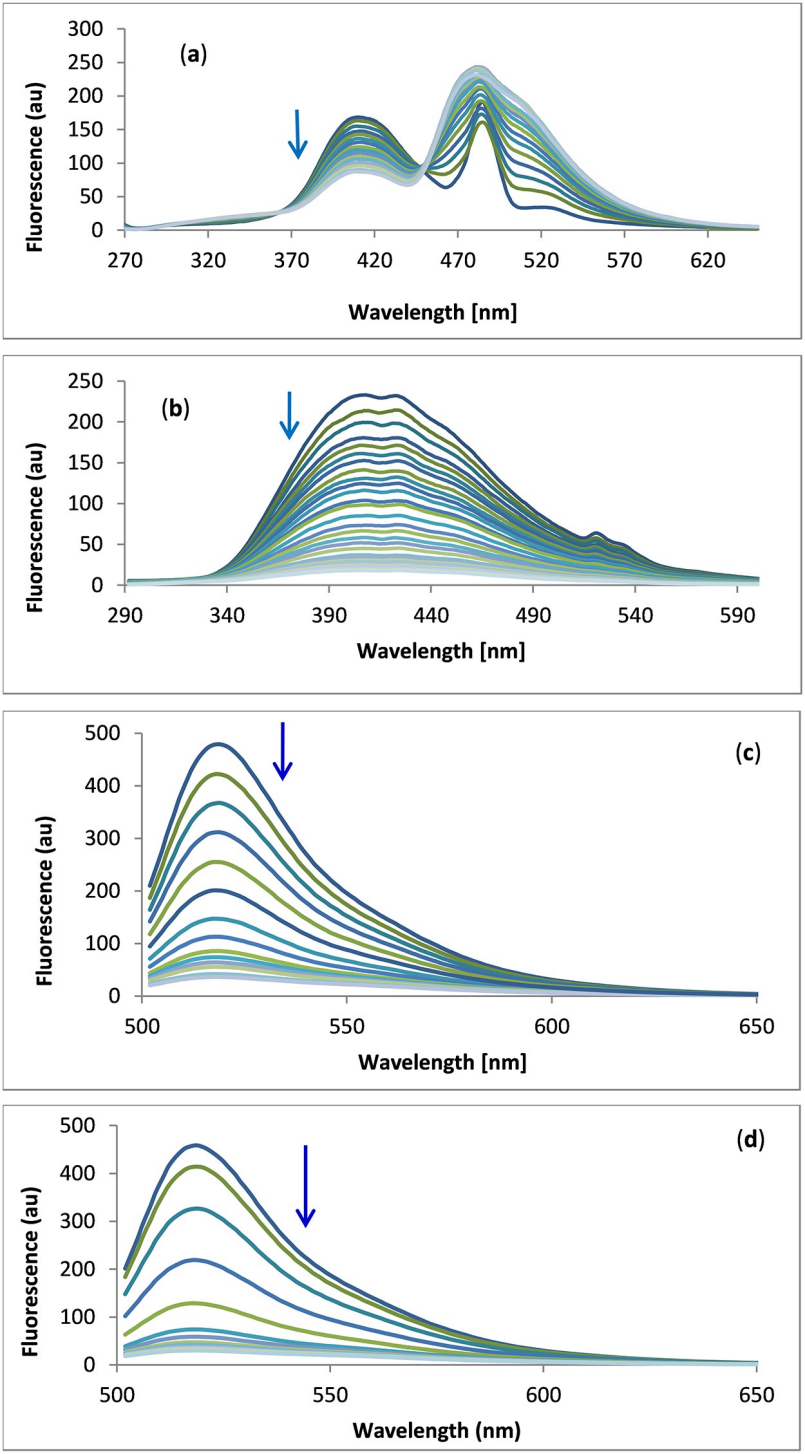
Fluorescence titrations of (a) A 5 × 10^−6^ M GW-2974 with human telomeric G-quadruplex DNA (1.33 × 10^−4^ M). (b) A 5 × 10^−6^ M of SCH-442416 with human telomeric G-quadruplex DNA (1.33 × 10^−4^ M). (c) A 2 × 10^−6^ M fluoresceine-labelled G-quadruplex DNA with 1 × 10^−4^ M GW-2974. (d) A 2 × 10^−6^ M fluorescein-labelled G-quadruplex DNA with 1 × 10^−4^ M SCH-442416. All titrations were carried out in Tris–KCl buffer, pH 7.4. Fluorescein-labelled G-quadruplex DNA had an excitation wavelength of 494 nm and emission wavelength of 518 nm.

The binding interactions of GW-2974 and SCH-442416 with human telomeric G-quadruplex DNA were confirmed through fluorescence titrations. Fig 2 shows the fluorescence spectra of GW-2974 and SCH-442416. The GW-2974 spectrum revealed a fluorescence band at 410 nm at excitation wavelength of 243 nm whereas the SCH-442416 spectrum revealed a broad fluorescence band centered at 420 nm at an excitation wavelength of 267 nm. The two bands can be attributed to π*-π transitions. The latter band appeared to be composed of two overlapping bands at 408 and 423 nm.

Sequential additions of G-quadruplex DNA to GW-2974 resulted in a continuous decrease in the fluorescence intensity at 410 nm. Additionally, a 65% hypochromicity was observed after adding 20 folds of G-quadruplex DNA (Fig 2a). The band at 484 nm is attributed to Rayleigh scattering, which is normally observed at a wavelength that is double the excitation wavelength. Similarly, adding G-quadruplex DNA to SCH-442416 continuously decreased the fluorescence emission at 420 nm (Fig 2b). Furthermore, when 20 folds of G-quadruplex DNA were added, the band showed a slight red shift associated with reduction in its intensity by 91.4% (hypochromic effect).

Additionally, reductions in fluorescence emissions of GW-2974 and SCH-442416 by ≥ 65%–91.4% revealed that these compounds can bind to human telomeric G-quadruplex DNA via intercalation binding modes [56].

#### Quenching of fluorescein labelled G-quadruplex DNA

More evidence on the intercalation of GW-2974 and SCH-442416 with telomeric G-quadruplex DNA was obtained from fluorescence quenching measurements. The fluorescein-labelled telomeric G-quadruplex DNA (5′-Fl-AGGG(TTAGGG)3-3′) displayed a high-intensity fluorescence band at 518 nm when excited at 494 nm. Sequential additions of GW-2974 or SCH-442416 (10^−4^ M) to fluorescein-labelled human telomeric G-quadruplex DNA (2 × 10^−6^ M) resulted in fluorescence quenching of more than 90% at 518 nm (Fig 2c and 2d). The quenching extent is dependent on the number of molecules bound per DNA molecule.

Additionally, the decreased fluorescence emission at 518 nm confirmed the intercalations of both the compounds at a location close to the fluorescein flag moiety. Since the fluorescein molecule is connected to the 5′ end, one may infer that both GW-2974 and SCH-442416 bind between the G-quartets adjacent to the TTA loop cavity.

These results are consistent with the previous results of UV–Vis absorption and fluorescence measurements, confirming the intercalation binding of GW-2974 and SCH-442416 with G-quadruplex DNA.

#### Circular dichroism

CD spectroscopy is the tool of choice for studying conformational changes in DNA caused by interactions with drugs. The structural conformation of human telomeric G-quadruplex DNA in solution is dependent on its sequence, length and environment. For example, they form an anti-parallel structure in Na^+^ solution, whereas in K^+^ solution, a hybrid parallel–anti-parallel structure is formed [70,71].

**Fig 3:**
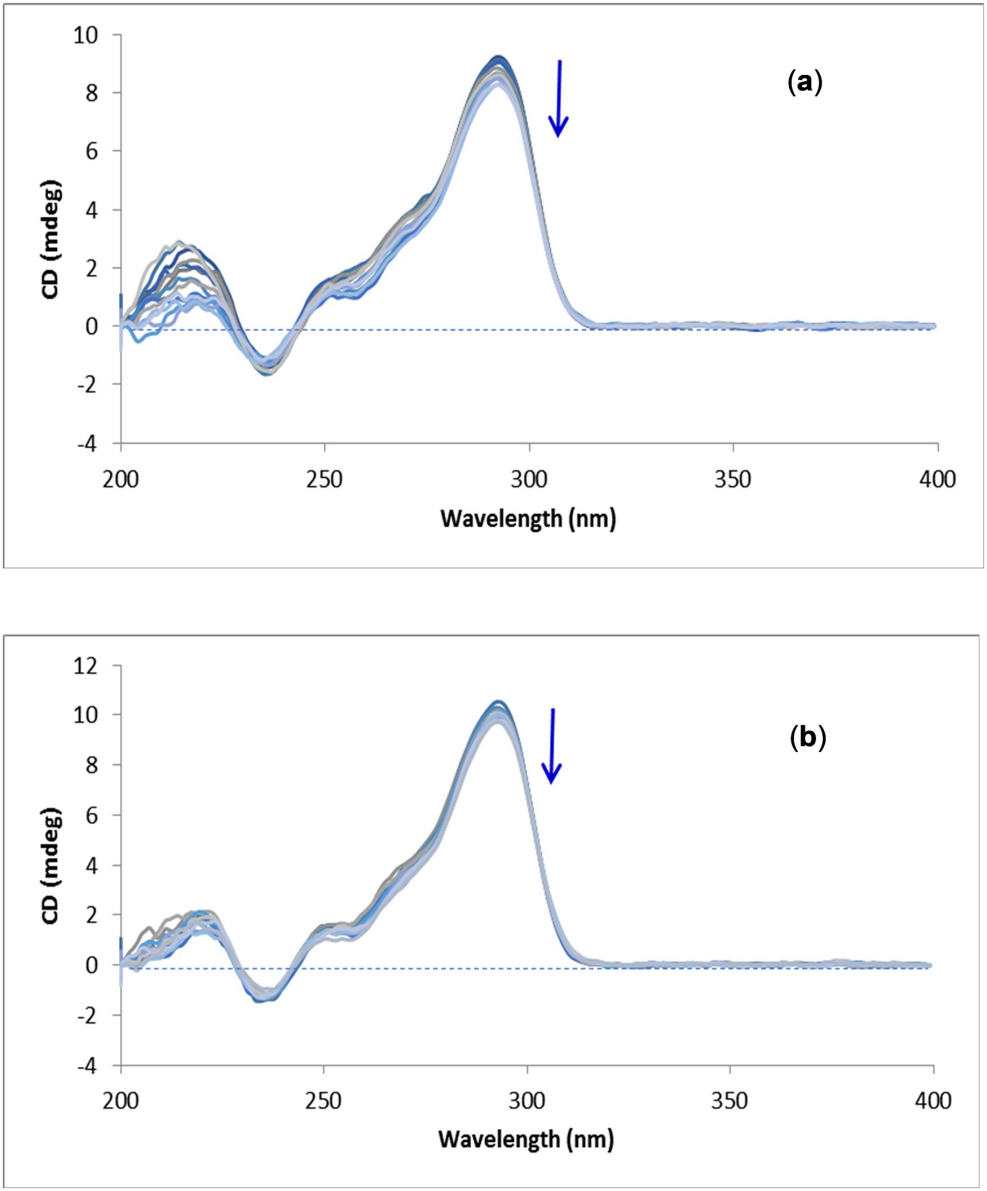
CD titration of human telomeric G-quadruplex DNA (1 × 10^−5^ M) with 1 × 10^−4^ M of GW-2974 (a) and SCH-442416 (b) in Tris–KCl buffer (pH 7.4).

Fig 3 illustrates the CD spectrum of the 22-base human telomeric G-quadruplex DNA in Tris–KCl buffer, pH 7.4. The presence of a positive band at 293 nm, a positive shoulder at 253 nm and a negative band at 235 nm confirmed the formation of a hybrid parallel–anti-parallel structure in K^+^ solution [61].

Fig 3a and 3b express the effects of adding GW-2974 and SCH-442416 to telomeric G-quadruplex DNA, respectively. However, the decreased band intensities at 235, 253 and 293 nm without alterations in the spectral shapes or bands’ positions showed no change in the hybrid parallel–anti-parallel conformation of the telomeric G-quadruplex DNA during the titration. Changes in the G-quadruplex DNA CD spectra during titration have been associated with the binding mode of the drug involved. Additionally, continual reduction in CD intensity was associated with the intercalation binding mode whereas the rise was linked with the groove binding mode [72].

Therefore, reductions in CD intensities observed on the sequential addition of GW-2974 or SCH-442416 could be linked with intercalation binding modes by via π-π stacking between the G-quartet faces of the G-quadruplex DNA (Fig 3). These results are also consistent with the previous results obtained for absorption and fluorescence measurements.

### 3.3. Binding stoichiometry

**Fig 4:**
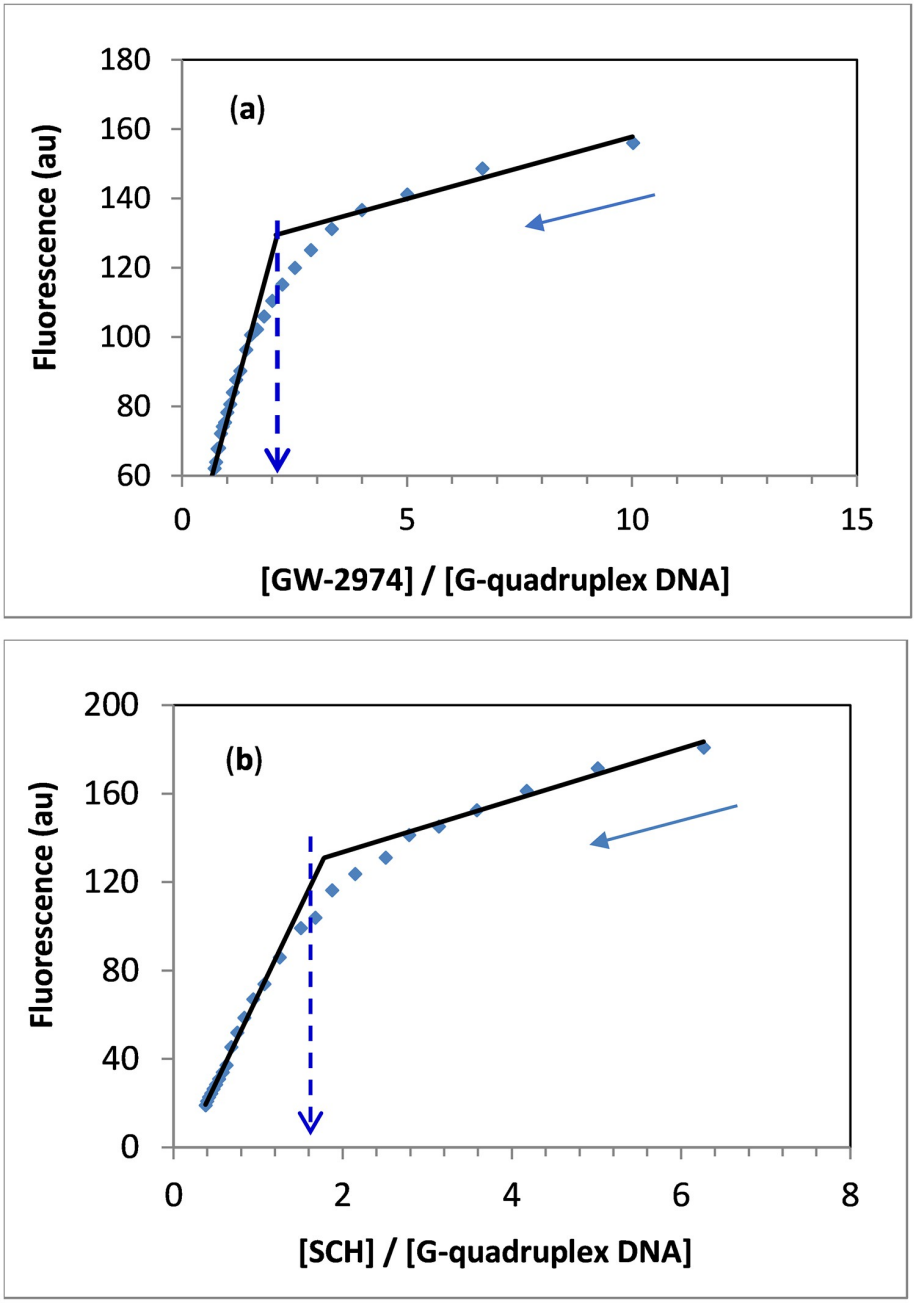
Stoichiometric ratio of [Drug]/{G-quadruplex DNA] using the molar ratio method. Hunan telomeric G-quadruplex DNA (1.44 × 10^−5^ M) was added in different increments to 2.0 ml of 5 × 10^−6^ M GW-2974 (**a**) or SCH-442416 (**b**). Fluorescence emission of the two compounds was followed at 410 or 420 nm, respectively. Measurements were carried out in Tris–KCl buffer, pH 7.4.

The molar ratio method based on fluorescence measurements was used to calculate the stoichiometry of the interactions of GW-2974 and SCH-442416 with human telomeric G-quadruplex DNA. The molar ratio curves obtained after adding increasing concentrations of telomeric G-quadruplex (1.44 × 10^−5^ M) to 2.0 mL of 5 × 10^−6^ M GW-2974 or SCH-442416 are shown in Fig 4. Results from the plots of fluorescence intensity at 410 and 420 nm versus molar ratio showed the molar stoichiometric ratios of GW-2974 and SCH-442416 to G-quadruplex DNA (Fig 4a and 4b). Additionally, a 2:1 molar ratio was obtained, indicating that two molecules of GW-2974 or SCH-442416 bound each G-quadruplex molecule. This ratio is consistent with the quercetin to human telomeric G-quadruplex DNA molar ratio [73].

### 3.4. Binding affinity

It has been suggested that there are several modes by which G-quadruplex DNA bind to drugs, including intercalation between adjacent G-quartets, π–π stacking on external G-quartet faces or within the loops and weak external binding [74].

Decreased intensity of the GW-2974 and SCH-442416 fluorescence soret bands after adding G-quadruplex DNA favored intercalations via face-to-face π–π stacking in the ratio 2:1 (GW-2974 or SCH-442416 to G-quadruplex DNA) (Fig 4).

**Fig 5:**
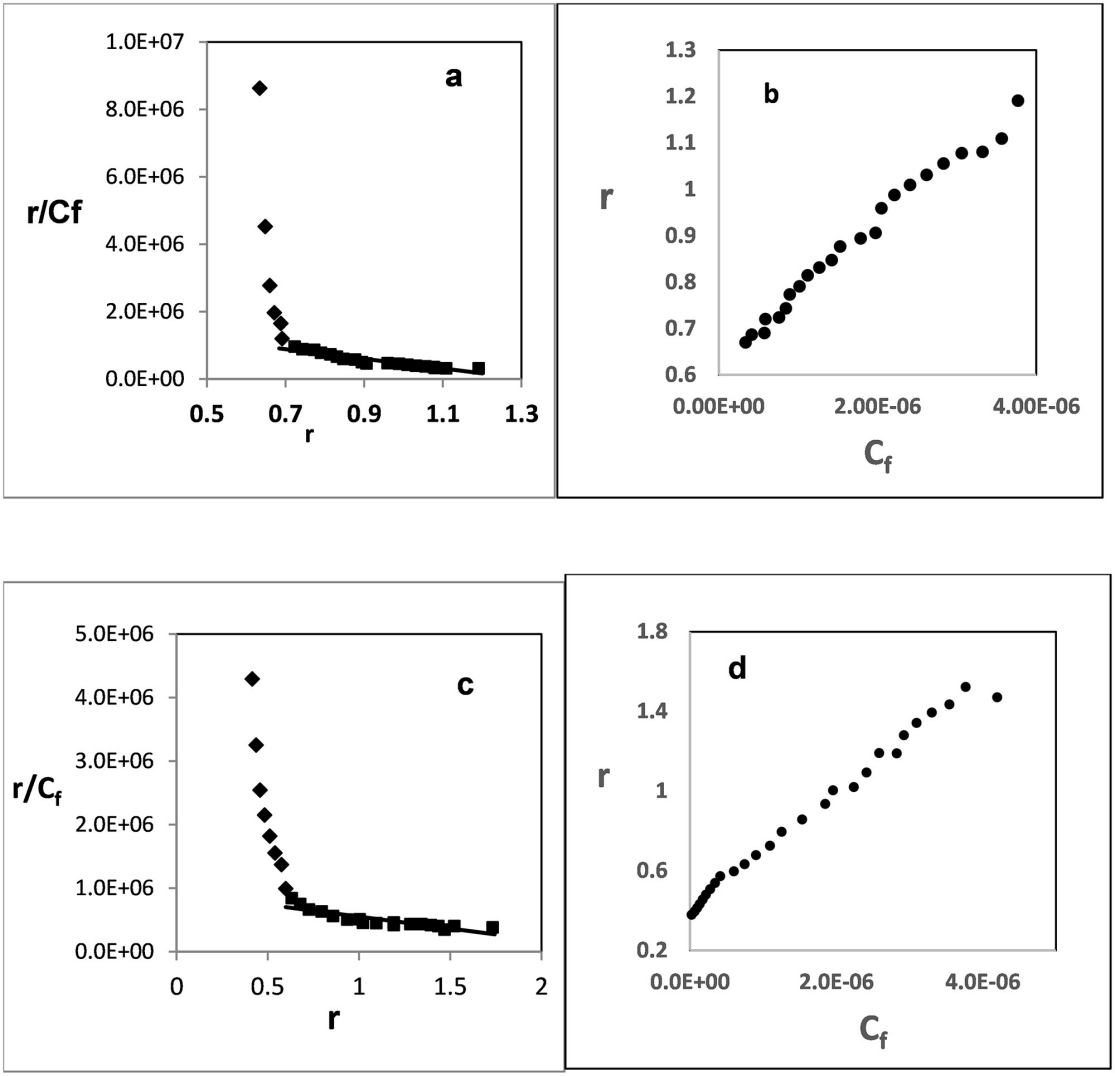
Nonlinear Scatchard plots. Increasing concentrations of human telomeric G-quadruplex DNA (1.44 x 10^−4^ M) were added to 2.0 ml of (5 × 10^−6^ M) GW-2974 (**a**, **b**) and SCH-442416 (**c**, **d**). The reaction was followed by measuring fluorescence emission of both compounds in Tris–KCl buffer, pH 7.4.

Additionally, the binding constants of the two compounds with G-quadruplex DNA were calculated using Scatchard plots. Fig 5 revealed non-linear downward concave curves of r/C_f_ versus r. These plots proposed that more than one type of dependent binding sites exist on the G-quadruplex molecule. Furthermore, dependent sites can synergistically or antagonistically affect themselves (neighbor exclusion effect). In the former, the first bound ligand encourages the subsequent binding ligand, whereas in the latter, the first bound ligand suppresses the subsequent binding ligand [75].

Resolving the non-linear Scatchard plots in Fig 5a and 5c produced two intersecting straight lines, representing two types of binding sites. The slopes of these lines resulted in binding constants of 1.3 × 10^8^ and 1.72 × 10^6^ M^−1^ for GW-2974 and 1.55 × 10^7^ and 3.74 × 10^5^ M^−1^ for SCH-442416. Additionally, the number of binding sites ranged between 0.7 and 1.27 for GW-2974 and 0.4 and 1.5 for SCH-442416 indicating a type of dependent binding sites (Fig 5a and 5c, Table 1).

**Table 1:** Binding constants (K) and number of binding sites (n) of GW-2974 and SCH-442416 per a G-quadruplex DNA molecule.

Complex formed	Site 1		Site 2	
	K1 (M^−1^)	n1	K2 (M^−1^)	n_2_
Linear curve fitting				
GW-2974—G-quadruplex	1.3 × 10^8^	0.7	1.72 × 10^6^	1.27
SCH-442416—G-quadruplex	1.55 × 10^7^	0.4	3.74 × 10^5^	1.5
Non-linear curve fitting				
GW-2974—G-quadruplex	2.65 × 10^6^	1.15	--	--
SCH-442416—G-quadruplex	7.0 × 10^5^	1.88	--	--

Application of the modified Scatchard Eq (5) normally yields a non-linear hyperbolic curve of r versus free ligand’s concentrations (Cf). Fig 5b and 5d show the plots of *r* versus Cf for the two investigated compounds. Semi-sigmoidal plots indicate the presence of more than one cooperative binding sites on the human telomeric G-quadruplex DNA molecule. Non-linear fitting of these plots revealed binding constants of 2.65 × 10^6^ and 7.0 × 10^5^ M^−1^ with 1.15 and 1.88 binding sites for GW-2974 and SCH-442416, respectively (Table 1).

Furthermore, these results reflected the high stabilization effects caused by binding both compounds on human telomeric G-quadruplex DNA. However, GW-2974 displayed higher binding affinities than SCH-442416. The number of binding sites was also consistent with that obtained through the molar ratio analysis and (Section 3.3).

### 3.5. Melting temperature

**Fig 6:**
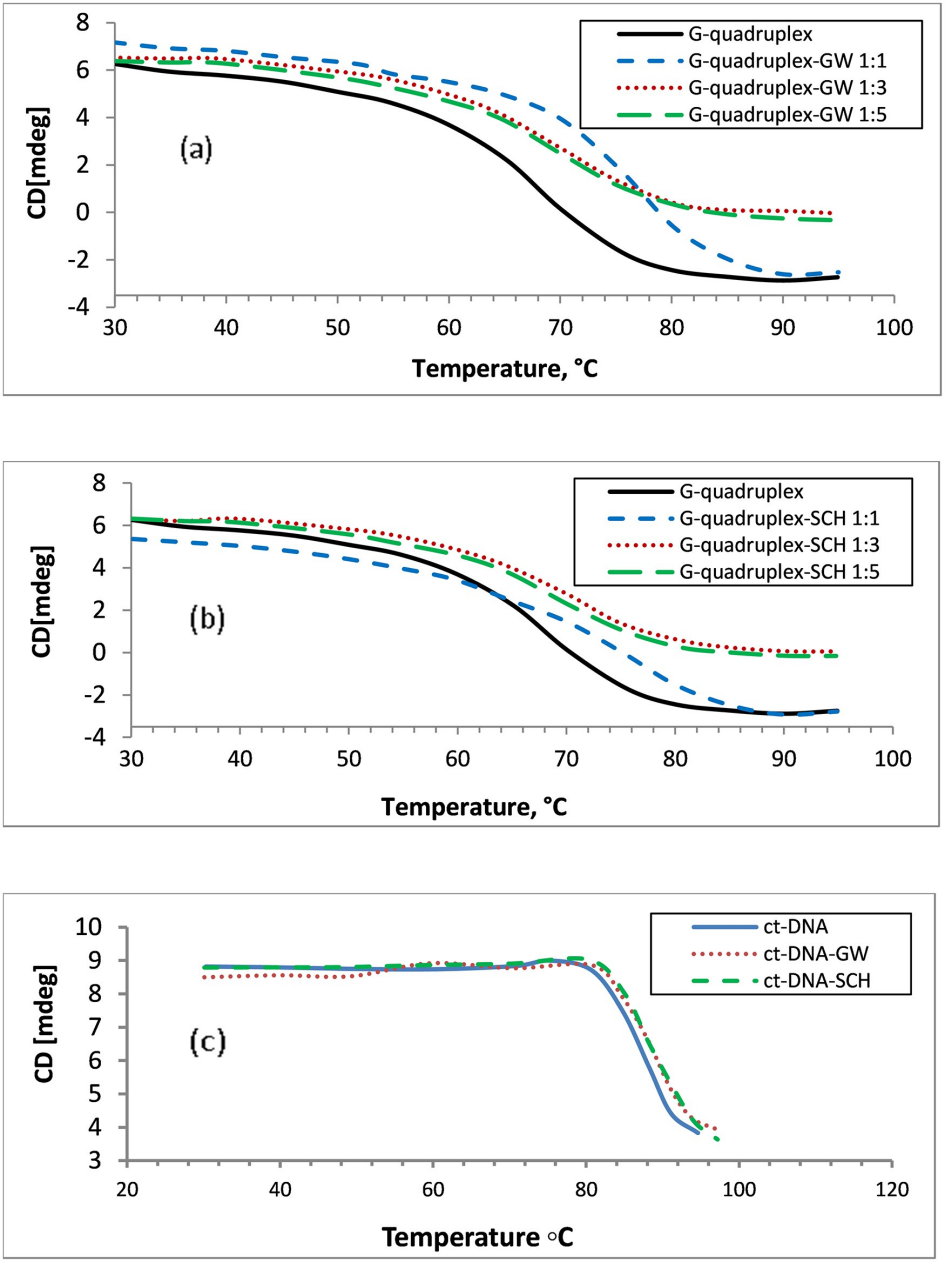
Melting temperature curves for human telomeric G-quadruplex DNA and its complexes with (**a**) GW-2974 and (**b**) SCH-442416 using 1:1, 3:1 and 5:1 [(Drug)/(G-quadruplex)] molar ratios. Equimolar concentrations of 3.93 x 10^−6^ M were used. Fig 6**c** shows the melting temperature curves for ct-DNA and its complexes with GW-2974 and SCH-442416. Equimolar concentrations of 1 x 10^−9^ M were used.

Fig 6 shows the T_m_ curves for G-quadruplex, ct-DNA and their complexes with GW-2974 and SCH-442416 based on CD measurements. T_m_ was calculated as the midway CD signal on the melting curve and represents the temperature where DNA is half unfolded into its single-stranded structure.

The T_m_ curve of human telomeric G-quadruplex DNA revealed a T_m_ value of 65.5°C, whereas its complexes with GW-2974 and SCH-442416 gave Tm values of 75.4°C and 75.1°C, respectively (Fig 6a and 6b) (Table 2). These results confirmed a stabilization effect of GW-2974 and SCH-442416 on G-quadruplex DNA with ΔTm = 9.9°C and 9.6°C, respectively. Additionally, these results revealed that GW2974 exerted a higher stabilizing effect than SCH-442416, which is consistent with the binding constants shown in Table 1. On the other hand, Fig 6c indicates a T_m_ of 88.0°C for ct-DNA. Additionally, complexations with GW-2974 and SCH-442416 stabilized the ct-DNA by Δ T_m_ values of 2.1°C and 2.4°C, respectively.

**Table 2:** Melting temperature of human telomeric G-quadruplex DNA and its GW-2974 and SCH-442416 complexes at different drug—DNA ratios. ΔT_m_ measures the difference between T_m_’s of the drug-DNA complex and pure DNA.

Tested compound	Drug: G-quadruplex molar ratio					
	1:1		3:1		5:1	
	T_m_ °C	ΔT_m_ °C	T_m_ °C	ΔT_m_ °C	T_m_ °C	ΔT_m_ °C
G-quadruplex DNA	65.5	0.0	65.5	0.0	65.5	0.0
GW-2974–G-quadruplex	75.4	9.9	69.7	4.2	69.0	3.5
SCH-442416–G-quadruplex	75.1	9.6	69.3	3.8	68.2	2.7
ct-DNA	88.0	0.0	--	--	--	--
GW-2974–ct-DNA	90.1	2.1	--	--	--	--
SCH-442416–ct-DNA	90.4	2.4	--	--	--	--

These results demonstrated that GW-2974 and SCH-442416 exerted a considerable stabilization effect (≥4-fold) on human telomeric G-quadruplex than on duplex ct-DNA (4.71 fold for GW-2974 and 4.00 fold for SCH-442416). The results also showed that both the compounds preferentially and selectively bind to G-quadruplex over duplex ct-DNA.

Fig 6 also illustrates the effect of GW-2974 and SCH-442416 concentrations on stabilizing human telomeric G-quadruplex DNA. Complexes with molar ratios of 1:1, 3:1 and 5:1 (drug:G-quadruplex) were evaluated for their T_m_ curves. Increasing the molar ratio of GW-2974 from 1:1 to 3:1 and 5:1 resulted in decreased ΔT_m_ values (from 9.9 °C to 4.2 °C and 3.5 °C, respectively). Similar effect were observed for SCH-442416 wherein ΔT_m_ values reduced from 9.6 °C to 3.8 °C and 2.7 °C when the molar ratio changed from 1:1 to 5:1 (Table 2). These results confirmed that increased ligands’ concentrations above 1:1 molar ratio decreased the stability of human telomeric G-quadruplex DNA. The reason could be attributed to the effect of molecular crowding caused by the excess GW-2974 or SCH-442416 molecules. Crowding effect was demonstrated to be dependent on the cation involved in the G-quadruplex’s formation and its topology [76].

Several molecules were tested for their crowding effects on G-quadruplex’s stabilization. These included polyethylene glycols (PEG), polysaccharides, ficoll and dextrans, ethanol, glycols, amino acids, acetonitrile, betaine, trimethylamine N-oxide and dimethyl sulfoxide (DMSO) [77 and refs. there in.]. High DMSO concentration decreased the ΔTm in hybrid telomeric G-quadruplexes DNA by 5–8 oC. Destabilization was also associated with conformation change from hybrid to parallel structures [77].

These reports support our findings that molecular crowding at high drugs’ concentrations could justify he observed decreases in G-quadruplex’s stabilization. Subsequently, our results suggested that the two investigated compounds stabilize human telomeric G-quadruplex DNA and possess high affinity and selectivity over duplex ct-DNA.

### 3.6. Binding selectivity

The selectivity of GW-2974 and SCH-442416 bindings to telomeric G-quadruplex DNA over duplex DNA was assessed using telomeric double-stranded DNA and ct-DNA as interfering species. Complexes produced by adding GW-2974 or SCH-442416 to fluorescein-labelled G-quadruplex DNA at 5 × 10^−10^ M each, were mixed with 10.0, 50.0 and 100.0 folds of telomeric duplex DNA or ct-DNA. Lastly, the mixtures were scanned for their fluorescence intensity at 518 nm as described in Section 2.4.5.

**Fig 7:**
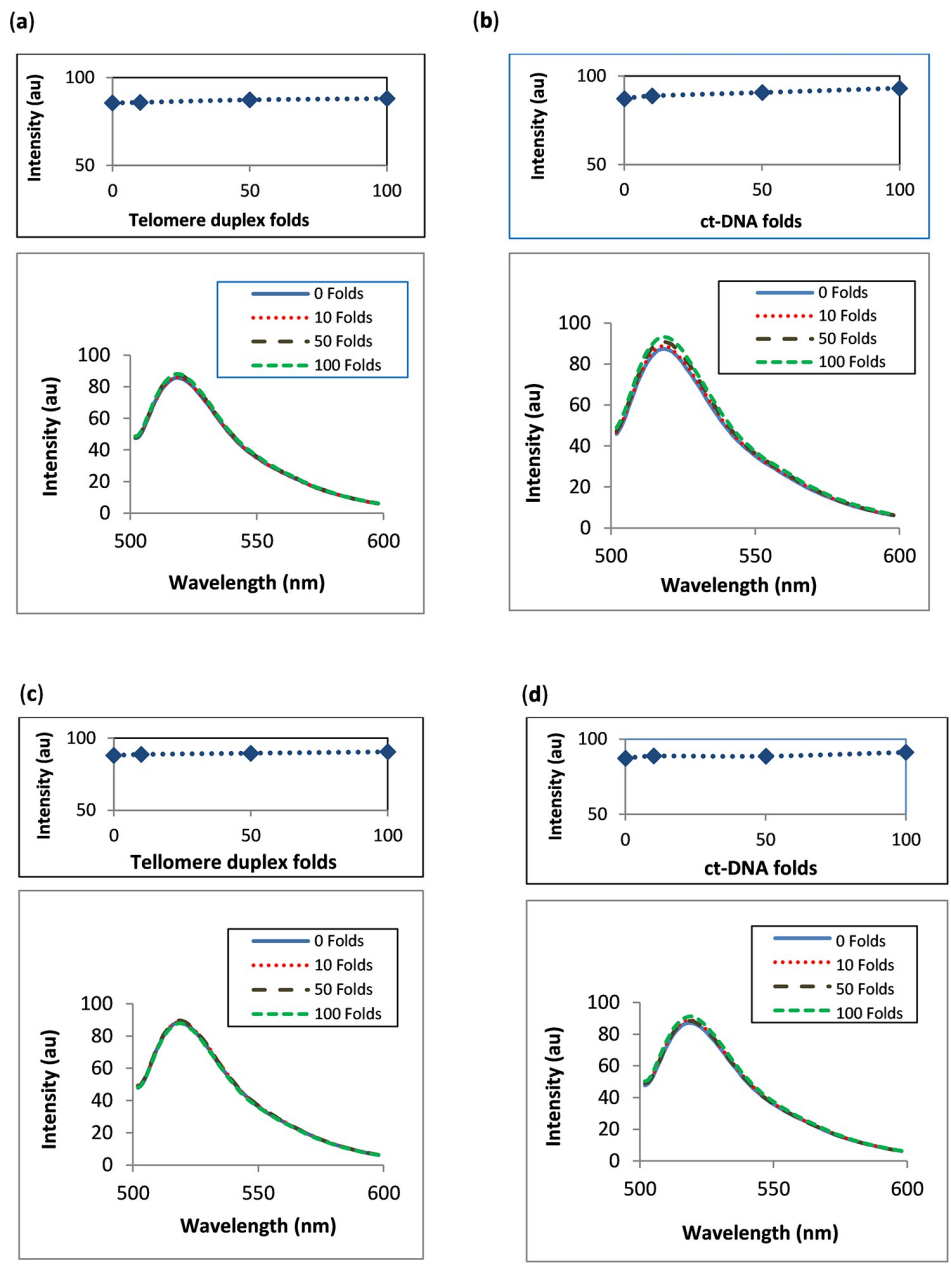
Selectivity of GW-2974 and SCH-442416 towards telomeric G-quadruplex DNA. Fluorescence of fluorescein-labelled G-quadruplex DNA (5 × 10^−10^ M) complexed with equimolar GW-2974 and SCH-442416 were measured in the presences of 10.0, 50.0 and 100.0 folds of telomeric double-stranded DNA (**a**, **c**) and ct-DNA (**b**, **d**). Measurements were performed in Tris–KCl buffer, pH 7.4.

Fig 7 shows the changes in the fluorescence intensity of fluorescein-labelled G-quadruplex complexes with GW-2794 and SCH-442416 upon adding different folds of telomeric double-stranded DNA (Fig 7a and 7c) and ct-DNA (Fig 7b and 7d). The selectivity coefficient was calculated by dividing the change in fluorescence intensity by the intensity of the fluorescein-labelled G-quadruplex complex. The coefficients with values of 1.1–2.2 × 10^−2^ and 2.2–4.4 × 10^−2^ were obtained. These results showed preferential and selective binding of GW-2974 and SCH-442416 to human telomeric G-quadruplex DNA over duplex double-stranded DNA and ct-DNA.

Therefore, the two compounds may selectively bind to G-quadruplex DNA over double-stranded DNA, which is the predominant DNA form in human cells. These results are also consistent with the results obtained from the T_m_ experiments (Section 3.5).

### 3.7. Molecular docking and dynamic simulations

#### Molecular docking

Several human telomeric G-quadruplex DNA structures are found on the protein data bank. Out of which, the (TTAGGGT)_4_ sequence form a parallel G-quadruplex structure in PDB:2MS6 and the (TTAGGG)_4_TT sequence form a hybrid G-quadruplex structure in PDB:6CCW in K+ ion solutions. In PDB:2MS6, quercetin drug is co-crystallized with G-quadruplex DNA on two binding sites whereas in PDB:6CCW, epiberberine drug is co-crystalized on one binding site. Both structures were solved by solution NMR and therefore, considered similar to investigated human telomeric G-quadruplex DNA ((AGGGTT)_3_AGGG) selected for this study.

Molecular docking of GW-2974 into the parallel G-quadruplex structure (PDB:2MS6) gave a docking score of −8.14 kcal/mol compared to −9.03 kcal/mol for the quercetin co-crystallized drug docked on the same pocket (Site 1, Table 3). Docking of SCH-44216 on the same site gave a score of -6.07 kcal/mol. On-site 2, GW-2974 and SCH-442416 presented almost similar binding scores (−6.17 and −6.37 kcal/mol, respectively), which are inferior to the score obtained by the co-crystallized quercetin (−9.27 kcal/mol). These results indicated high binding affinities for both compounds to parallel G-quadruplex DNA.

**Table 3:** Docking scores of GW-2974, SCH-442416 and the co-crystallized drugs (quercetin and epiberberine) into the binding sites of the parallel (2MS6) and the hybrid (6CCW) human telomeric G-quadruplex DNA structures.

Drug		Docking score (kcal/mol)		
		2MS6		6CCW
		Site 1	Site 2	--
GW-2974		−8.14	−6.17	−6.28
SCH-442416		−6.07	−6.37	−4.77
Co-crystallized ligand	Quercetin	−9.03	−9.72	--
	Epiberberine	--	--	−4.27

**Fig 8:**
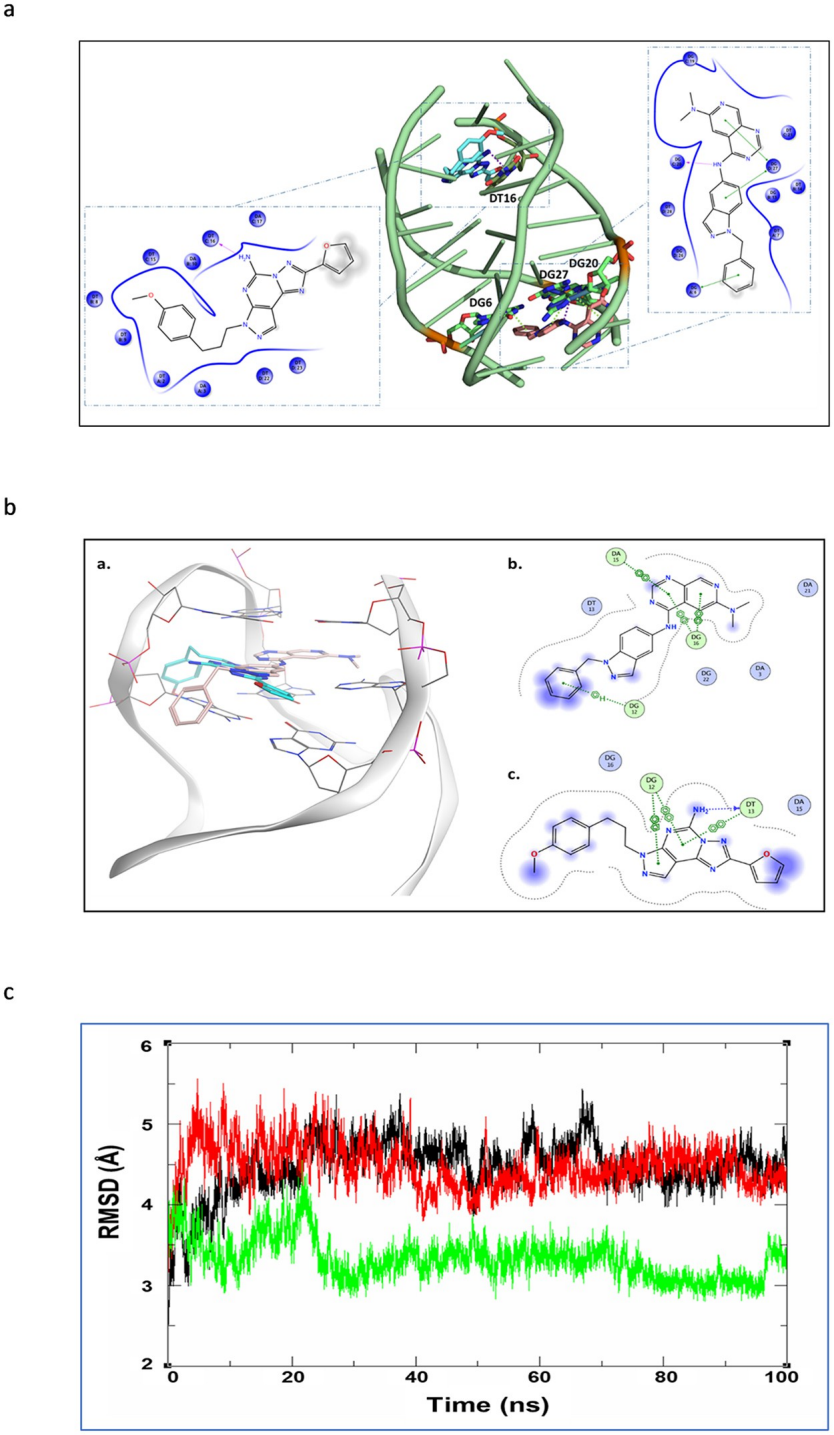
**a. Docking of GW-2974 and SCH-442416 into the binding sites of the parallel human telomeric G-quadruplex DNA structure (2MS6)**. Predicted binding modes of GW-2974 (pink sticks) and SCH-442416 (blue sticks) on G-quadruplex DNA (green sticks and cartoon). The 2D ligand interaction diagram shows the hydrogen bonding and π–π interaction as purple and green dotted lines, respectively. **b. Docking of GW-2974 and SCH-442416 into the binding sites of the hybrid telomeric G-quadruplex DNA structure (6CCW)**. The G-quadruplex DNA with the two docked compounds is shown in **(a)**. The 2D interaction diagrams show the binding modes of GW-2974 as pink sticks **(a)** and of SCH-442416 as green sticks in **(b)**. **c. The RMSD analysis of the docked complexes of the** Telomeric G-quadruplex DNA with GW2974 (black), SCH-442416 (red), and the co-Crystallized ligand (green).

Fig 8a shows a cartoon representation for docked GW-2974 and SCH-442416 onto the parallel G-quadruplex structure (PDB:2MS6). The two compounds were able to slide between the nitrogen bases surrounding both binding sites 1 and 2. On site 1, GW-2974 bound to DG20 via hydrogen bonding and to DG6 and DG27 via multiple stacking interactions. On site 2, SCH-442416 interacted with DT16 via hydrogen bonding, as important interaction.

Docking of GW-2974 and SCH-442416 onto the hybrid G-quadruplex structure (PDB:6CCW) is represented in Fig 8b(a). Favorable exothermic bindings with docking scores of −6.28 and -4.77 kcal/mol were respectively obtained. The co-crystallized drug epiberberine docked on the same site gave less binding energy of −4.27 kcal/mol (Table 3). Fig 8b(b) shows a 2D interaction diagram. GW-2974 was able to slide between the nitrogen bases surrounding the binding site and to make hydrogen bonding with DG20 along with multiple stacking interactions with DG12, DA15 and DG16. The diagram 8b(c) shows that SCH-442416 was also able to slide between the nitrogen bases of DG12 and DT13 and to make hydrogen bonding with DT13.

To summarize, our docking results are in line with the experimental data, suggesting that GW-2974 and SCH-442416 favorably and exothermally bind to the parallel and hybrid structures of human telomeric G-quadruplex DNA. On the parallel structure, the two compounds conveniently docked into the two quercetin binding sites forming 2:1 (drug:G-quadruplex) complexes with binding energies of -8.14 to -6.17 kcal/mol for GW-2974 and -6.37 to -6.07 kcal/mol for SCH-442416.

Epiberberine was reported to specifically recognize the hybrid-2 form of telomeric G-quadruplex and to convert other telomeric structures (e.g. hybrid-1 and basket-type) into the hybrid-2 structure. In K^+^ solution, epiberberine induces an extensive four-layer binding pocket specific to hybrid-2 form of telomeric G-quadruplex DNA [78]. Docking of GW-2974 and SCH-442416 into the epiberberine binding pocket revealed exothermic bindings (−6.28 and -4.77 kcal/mol, respectively) relative to −4.27 kcal/mol of epiberberine.

Additionally, our experiments revealed that both GW-2974 and SCH-442416 form 2:1 (drug:G-quadruplex) complexes on contrary to the 1:1 complex species reported for epiberberine. The reason may be attributed to the neutrality and flexibility of both molecules relative to the rigidity and cationic character of epiberberine.

### Molecular dynamics

Stability of human telomeric G-quadruplex complexes with GW-2974 and SCH-442416 were assessed using a 100 ns molecular dynamic simulation on docked poses. Fig 8c shows that the RMSD curves of both complexes have converged quickly by the 20 ns time-step and remained slightly oscillating around 4.5 Å till the end of the simulation. The epiberberine co-crystallized complex showed similar behavior with lower RMSD values oscillate around 3.5 Å.

The GW-2974 and SCH-442416 complexes showed a greater shift from the starting conformation (indicated by the higher RMSD values) as they needed to adjust themselves to find the best fitting. On contrary, the smaller shift in epiberberine complex can be attributed to capability of the co-crystallized ligand itself to induce the groove in the targeted DNA structure.

Subsequently, the above results of docking and MD simulations suggest that both GW-2974 and SCH-442416 have the potential to bind and stabilize human telomeric G-quadruplex DNA, which comes in line and support our experimental findings.

## 4. Conclusions

GW-2974 is a highly potent tyrosine kinase receptor that inhibits EGFR and ERBB-2 in tumor cells with selectivity for tumor cells over normal cells. SCH-442416 is a potent adenosine A_2A_ receptor antagonist with high selectivity for human A_2A_ receptors. Various anti-cancer effects and mechanisms of these compounds have been documented in the literature.

In this study, human telomeric G-quadruplex DNA was evaluated as an additional molecular target through which both the compounds exert their anti-cancer effects. Additionally, changes in UV–Vis spectra, fluorescence spectra, CD spectra and melting temperature as well as *in-silico* molecular docking were used to provide evidence for their interactions with human telomeric G-quadruplex DNA.

The changes in UV–Vis and fluorescence spectra showed that GW-2974 and SCH-442416 bind to human telomeric G-quadruplex DNA via intercalation. Furthermore, fluorescence quenching of fluorescein-labelled G-quadruplex DNA observed after adding the two compounds indicated that they bound at a location close to the fluorescein flag, i.e., close to the TTA loop. Additionally, decreased CD intensities at 235, 253 and 293 nm indicated the presence of intercalation bindings.

Estimations of binding affinities using Scatchard plots revealed non-linear downwards concave plots. Graphical resolution of these plots indicated the presence of two types of dependent binding sites with binding constants of 1.3 × 10^8^ and 1.72 × 10^6^ M^−1^ for GW-2974 and 1.55 × 10^7^ and 3.74 × 10^5^ M^−1^ for SCH-442416. The corresponding number of binding sites were 0.7–1.27 and 0.4–1.5 per G-quadruplex DNA molecule, respectively. Non-linear curve fitting resulted in average binding constants of 2.65 × 10^6^ and 7.0 × 10^5^ M^−1^ with 1.15 and 1.88 binding sites for GW-2974 and SCH-442416, respectively. The molar ratio method indicated that GW-2974 and SCH-442416 form 2:1 complexes with telomeric G-quadruplex DNA. The T_m_ curves showed that GW-2974 and SCH-442416 stabilized human telomeric G-quadruplex DNA by 9.6 °C and 9.1 °C, respectively; this result is more than four times higher than that obtained for double stranded ct-DNA (2.1 °C and 2.4 °C, respectively).

Selectivity experiments produced coefficients ≤ 3.0 × 10^−2^, revealing preferential bindings for both compounds to human telomeric G-quadruplex DNA over the double stranded telomeric and calf thymus DNA. This conclusion was confirmed by the obtained ΔT_m_ values that were 4.7- and 4.0-fold higher than the ΔT_m_ values for ct-DNA. Furthermore, *in silico* molecular docking and dynamic simulation indicated stable and favorable exothermal binding on binding sites of G-quadruplex parallel and hybrid structures. Binding scores comparable to the co-crystallized drug quercetin and better than epiberberine were obtained and confirmed our experimental results. Furthermore, GW-2974 had a higher affinity and ΔT_m_ values than SCH-442416. Since G-quadruplex DNA bind small molecules via intercalation between adjacent G-quartets, end-stacking or π-π stacking, it could be inferred from the above results that GW-2974 and SCH-44241 have intercalated between adjacent G-quartets (π-π* stacking) and end-stacking on G-quartets [74].

In summary, our results suggest that the two investigated compounds can act selectively as human telomeric G-quadruplex DNA stabilizers. Selective recognition and stabilization’s abilities could be responsible for their anti-cancer effects.

## Supporting information

S1 File CD spectrum of 2.36 × 10^−6^ M telomeric G-quadruplex in Tris–KCl buffer (pH 7.4) titrated with ethylene glycol (0–200 μL). (DOCX)

S2 File CD spectrum of human telomeric G-quadruplex (4 × 10^−6^ M) in Tris–KCl buffer (pH 7.4). (DOCX)

S3 File (XLSX)

S4 File (XLSX)
