# Supplementary material for: GW-2974 and SCH-442416 modulators of tyrosine kinase and adenosine receptors can also stabilize human telomeric G-quadruplex DNA
Source: PLoS One. 2022 Dec 7;17(12):e0277963. doi: 10.1371/journal.pone.0277963 (PMC9728906; doi:10.1371/journal.pone.0277963)
Supplement: S1 File — (DOCX) [file pone.0277963.s001.docx]

S1


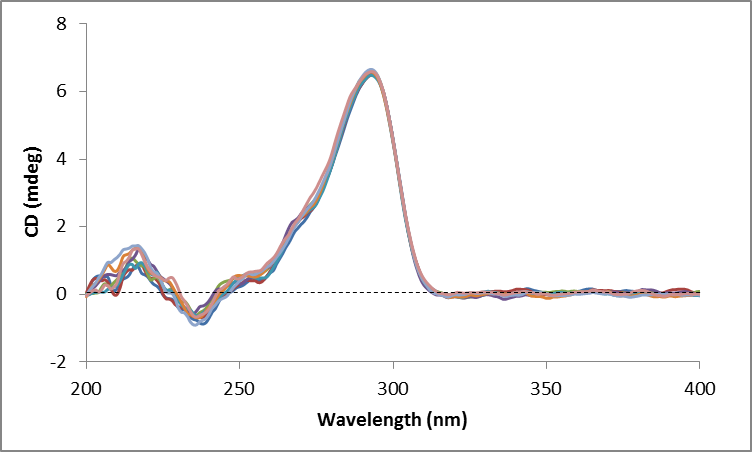


S1. CD spectrum of 2.36 × 10^−6^ M telomeric G-quadruplex in Tris–KCl buffer (pH 7.4) titrated with ethylene glycol (0–200 µL).
