# Supplementary figures and images for: GW-2974 and SCH-442416 modulators of tyrosine kinase and adenosine receptors can also stabilize human telomeric G-quadruplex DNA
Source: PLoS One. 2022 Dec 7;17(12):e0277963. doi: 10.1371/journal.pone.0277963 (PMC9728906; doi:10.1371/journal.pone.0277963)

S2


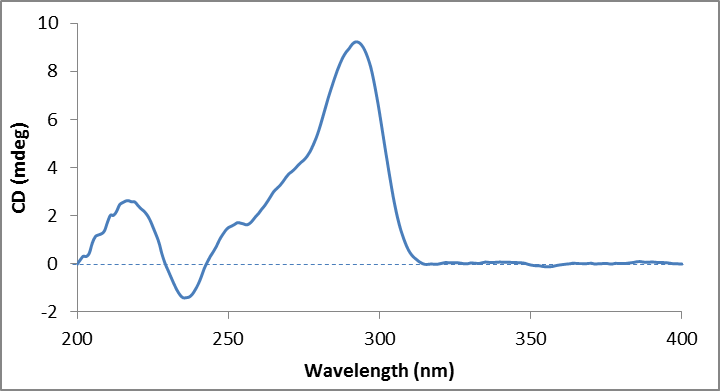


S2. CD spectrum of human telomeric G-quadruplex (4 × 10−6 M) in Tris–KCl buffer (pH 7.4).

Supplement: S2 File — (DOCX) [file pone.0277963.s002.docx]
